# Cross-sectional epidemiological investigations of *Giardia lamblia* in children in Pakistan

**DOI:** 10.1590/1516-3180.2018.0350060918

**Published:** 2018-10-22

**Authors:** Aneeqa Naz, Zeeshan Nawaz, Muhammad Hidayat Rasool, Muhammad Asif Zahoor

**Affiliations:** I BSc, MSc. Microbiologist and Doctoral Student, Department of Microbiology, Government College University Faisalabad, Faisalabad, Pakistan.; II MSc, PhD. Microbiologist and Assistant Professor, Department of Microbiology, Government College University Faisalabad, Faisalabad, Pakistan.; III MSc, PhD. Microbiologist and Associate Professor, Department of Microbiology, Government College University Faisalabad, Faisalabad, Pakistan.; IV MSc, PhD. Microbiologist and Assistant Professor, Department of Microbiology, Government College University Faisalabad, Faisalabad, Pakistan.

**Keywords:** Prevalence, Giardia lamblia, Risk factors, Child, Pakistan

## Abstract

**BACKGROUND:**

The prevalence of *Giardia lamblia* in Pakistani children is currently unknown. The aim here was to evaluate the prevalence and risk factors of *Giardia lamblia* in children exhibiting diarrhea.

**DESIGN AND SETTING:**

Cross-sectional study at different district healthcare hospitals in Pakistan.

**METHODS:**

A total of 800 samples were collected from children aged 0-10 years. Information regarding personal data, demographic data and supposed risk factors was collected through a structured questionnaire. *Giardia lamblia* was detected through direct microscopy and antigens through the enzyme-linked immunosorbent assay (ELISA).

**RESULTS:**

The prevalence of *Giardia lamblia* was 2.75% through direct microscopy and inflated to 9.5% through ELISA. The demographic factors positively associated with occurrences of giardiasis were age (P = 0.035; odds ratio, OR = 1.96; 95% confidence interval, CI = 1.094-3.533), mother’s educational level (P = 0.031; OR = 2.67; 95% CI = 1.186-6.045) and father’s educational level (P = 0.004; OR = 3.56; 95% CI = 1.612-7.899). Similarly, among the supposed risk factors, rural residency (P = 0.032; OR = 1.76; 95% CI = 1.098- 2.851), absence of proper sewerage system (P = 0.000; OR = 6.60; 95% CI = 4.029-10.841) and unavailability of safe drinking water (P = 0.000; OR = 4.08; 95% CI = 2.207-7.547) were the factors strongly connected with giardiasis. Abdominal discomfort was a prominent clinical sign with 46% frequency.

**CONCLUSION:**

Various risk factors were associated with occurrences of *Giardia*, thus emphasizing the importance of parents’ education, safe drinking water and proper sewerage systems for Pakistani children’s health.

## INTRODUCTION

*Giardia lamblia*, which is also recognized as *Giardia intestinalis* or *Giardia duodenalis*, is the most common protozoon infecting the small intestine of humans and is a major cause of enteric infection throughout the world, especially in children.^1^*Giardia* was first reported by a scientist named Leeuwenhoek in his own stools, in 1681.^2^*G. lamblia* is the only known species of *Giardia* found in humans and other mammals. Seven different genotypes/assemblages (A to G) of *Giardia lamblia* with host specificity have been reported. Assemblages A and B have been reported in humans, cattle and many other mammals.^3^ The typical signs of giardiasis include diarrhea, malaise, greasy stools, flatulence, abdominal cramps, bloating and weight loss.^4^ Depressed levels of intestinal enzymes and disaccharides are observed, along with absorption defects regarding fat, lactose, vitamin A and vitamin B12.^5^

The life cycle of *Giardia lamblia* involves two stages: trophozoite and cyst. Trophozoites are responsible for producing clinical disease in humans by attaching themselves to the walls of the small intestine, followed by rapid multiplication. On the other hand, cysts are infectious in nature and are responsible for disease transmission. After ingestion of cysts, excystation occurs in the proximal part of the small intestine, resulting in release of trophozoites.^6^*Giardia* cysts are the environmentally stable stage and are resistant to inactivation by various water disinfectants, which makes them viable for up to two months.^3^ Transmission of *Giardia* occurs mainly through contaminated water and food. Other factors involved in this include poor living conditions, overcrowded housing, poor environmental sanitation, unhygienic personal habits, unsafe water supply and low socioeconomic class.^7^

The preliminary diagnosis is based on clinical signs presented by children, and it is confirmed through detection of cysts and trophozoites in stool samples by means of direct observation under a microscope, which is considered to be the gold standard for diagnosing *Giardia*.^8^ Antigens in stool samples are detected through the enzyme-linked immunosorbent assay (ELISA), which is currently the most sensitive and most frequently used technique.^9^ The prevalence of *Giardia lamblia* is variable: in developed areas of the world, it ranges from 2% to 5%;^10^ while in developing countries, the prevalence level is quite high. A major part of this prevalence consists of children under 10 years of age, particularly those who are malnourished.^11^

Although *Giardia lamblia* is considered to be a common zoonotic intestinal parasite in children and adults in Pakistan, the current picture regarding prevalence of *Giardia* in children in Pakistan is still unclear.

## OBJECTIVES

The objectives of the present study were to estimate the prevalence of *Giardia lamblia* and identify its possible associated risk factors among children in Pakistan. This was a comprehensive study conducted on children in Pakistan, to assess the predisposing factors for *Giardia* infection.

## METHODS

### Participants, stool sample collection and settings

Stool samples were collected from children aged 0-10 years exhibiting diarrhea, by means of non-probability-based convenience sampling, between July 2016 and July 2017. The samples were collected from eight healthcare centers (two from each district) located in different districts of Pakistan, including Faisalabad, Khanewal, Multan and Rawalpindi.

A structured questionnaire containing dichotomous questions was designed and was presented to each parent at the time of sample collection from the respective child. The purpose of the questionnaire was to gather information regarding personal details, demographic data and supposed risk factors such as housing and living conditions, contacts with pets and parents’ educational level.

A stool sample of 5-10 grams was collected from each child in a sterile plastic container by a trained hospital staff member and was labeled properly. The samples were mixed with 10% formalin and were placed in refrigerator until they were transported to the laboratory, which was done within 24 hours after collection. About half of each sample was used for direct examination under a microscope and the remainder was stored at -80 °C to be used for antigen detection.

### Ethical approval

This study was approved by our Institutional Ethics Review Committee under code GCUF/ERC/4155 on April 25, 2016, and the samples were collected in accordance with international safety rules and ethical standards. Written consent was obtained from each parent after they had been given explanations regarding the purpose and objectives of the study.

### Detection of *Giardia* in stool samples

The sample obtained from each child was fixed with formalin and a wet-mount was prepared to detect any presence of *Giardia lamblia* (cysts or trophozoites) in the form of a direct smear, using 5% Lugol’s iodine and the concentration method.^12^

### Detection of *Giardia lamblia* antigen in stool samples

The stool material was subjected to the Ridascreen *Giardia* enzyme immunoassay (R-Biopharm AG, Germany) to detect *Giardia lamblia* antigens, in accordance with the manufacturer’s instructions.^13^ Positive and negative controls were also run, using test samples. Optical density (OD) was measured using an ELISA reader (Bio-Rad iMark, USA). Positive results were indicated as OD readings that were 10% over the cutoff value, as described in the manufacturer’s instructions.

### Statistical analysis

The data obtained were tabulated in a Microsoft Excel spreadsheet and were analyzed using STATA version 12 (Stata Corp., USA). Descriptive analysis was used to summarize the data on the basis of percentages and chi-square tests. In the present study, stool samples were considered positive for *Giardia lamblia* if any of the tests were positive.

Bivariate analysis was conducted to establish associations between risk factors and presence of giardiasis in children. Odds ratios (OR) were calculated at 95% confidence intervals (CI).^9^ P-values < 0.05 were considered to be statistically significant.

## RESULTS

The present study involved 800 children who were evaluated during the study period due to diarrhea, comprising 412 males (51.50%) and 388 females (48.50%) ranging in age from 0 to 10 years. On the basis of age, the children were divided into two groups: 0-5 years (n = 549) and 6-10 years (n = 251). 

The results from our study showed that out of the total of 800 samples, 22 (2.75%) were positive for giardiasis according to the direct method under a microscope. The ELISA test showed prevalence of 8.88%, through detecting *Giardia lamblia* in 71 samples. Five samples were found to be negative according to ELISA but were positive through direct examination under a microscope. Similarly, 30 samples were found to be negative through direct examination but were positive according to ELISA. Thus, the overall prevalence of *Giardia lamblia* infection was 9.5%, i.e. 76/800 samples were positive.

The prevalence of *Giardia lamblia* was non-significantly different on the basis of geographical location (P = 0.278). It was found to be highest (12.5%) in the Khanewal district, while the lowest prevalence was observed in the Faisalabad district (6.5%), as shown in Table 1. On the basis of area of residence, it was recorded that children living in rural areas were more prone to *Giardia lamblia* infection than were those living in urban areas. Statistically, these results were also significant [P = 0.032; OR = 1.76; 95% CI = 1.098- 2.851]. Conversely, children living in houses with proper sewerage and drainage systems were well protected from this infection [P = 0.000; OR = 6.60; 95% CI = 4.029-10.841], as also were those who had the facility of proper drinking water and a water supply system, who also had very small chances of getting this infection [P = 0.000; OR = 4.08; 95% CI = 2.207-7.547].

**Table 1.** Prevalence of *Giardia lamblia* among children in different districts in Pakistan

Area	Male		Female		Total	
	Total sampled	Total positive (percentage)	Total sampled	Total positive (percentage)	Total sampled	Total positive (percentage)
Faisalabad	104	8 (7.7%)	96	5 (5.2%)	200	13 (6.5%)
Multan	109	13 (11.9%)	91	8 (8.8%)	200	21 (10.5%)
Khanewal	101	17 (16.8%)	99	8 (7.8%)	200	25 (12.5%)
Rawalpindi	98	9 (9.2%)	102	8 (8.1%)	200	17 (8.5%)
Total	412	47 (11.40%)	388	29 (7.5%)	800	76 (9.5%)

On the basis of gender, the prevalences of giardiasis in male and female children were 11.40% (47/412) and 7.5% (29/388), respectively. Statistically, there was no significant variation in the prevalence of *Giardia* with regard to gender (P > 0.05).

A significant difference in the prevalences of *Giardia lamblia* was found between the children in the two age groups. The rate of susceptibility to giardiasis was higher (11.11%) among the children in the age range 0-5 years than among those in the age range 6-10 years (5.97%) [P = 0.035; OR = 1.96; 95% CI = 1.094-3.533], as shown in Table 2.

**Table 2.** Prevalence of *Giardia lamblia* among children, according to demographic characteristics

Risk factors	n	Positive (%)	P-value	Odds ratio	95% confidence interval
Gender (n = 800)					
Male Female	412 388	47 (11.40%) 29 (7.50%)	0.084	1.59	(0.981-2.589)
Age (n = 800)					
0-5 years 6-10 years	549 251	61 (11.11%) 15 (5.97%)	0.035	1.96	(1.094-3.533)
Birth order (n = 788)					
Not first First	73 715	8 (10.95%) 68 (9.51%)	0.718	1.17	(0.539-2.543)
No. of children (n = 800)					
More than one	355 445	27 (7.60%) 49 (11.01%)	0.137	1.50	(0.919-2.458)
Mother’s education (n = 785)					
Not educated Educated	42 743	8 (19.05%) 60 (8.08%)	0.031	2.67	(1.186-6.045)
Father’s education (n = 763)					
Not educated Educated	38 725	9 (23.70%) 58 (8.00%)	0.004	3.56	(1.612-7.899)
Day care center (n = 800)					
Yes No	188 612	21 (11.17%) 55 (8.99%)	0.419	1.27	(0.748-2.167)

The parents’ educational level was significantly associated with the prevalence of giardiasis among children. The children of uneducated mothers were more likely to have *Giardia lamblia* infection than were those of educated mothers [P = 0.031; OR = 2.67; 95% CI = 1.186-6.045]. Similarly, children whose fathers had not had any education were 3.5 times more at risk of being infected with *Giardia lamblia* than were those whose fathers had received education [P = 0.004; OR = 3.56; 95% CI = 1.612-7.899], as shown in (Table 2).

The percentages were found to be variable among the groups of different suspected risk factors, but statistically there was no association between the prevalence of giardiasis and some variables like birth order, number of children, attendance at a day care center, living in a house versus in an apartment, availability of washrooms and presence of pets at home (Table 3). The predominant clinical presentations among the children infected with *Giardia lamblia* were abdominal pain and discomfort (46%), vomiting (13.15%) and bloody diarrhea (10.52%).

**Table 3.** Prevalence of *Giardia lamblia* among children, according to housing characteristics

Risk factors	n	Positive (%)	P-value	Odds ratio	95% confidence interval
Residence (n = 800)					
Rural Urban	350 450	43 (12.28%) 33 (7.33%)	0.032	1.769	(1.098-2.851)
Housing (n = 800)					
Apartment House	285 515	31 (10.87%) 45 (8.73%)	0.370	1.274	(0.787-2.064)
Sewerage system (n = 800)					
Yes No	650 15	35 (5.38%) 41 (27.33%)	0.000	6.604	(4.029-10.841)
Bathroom (n = 800)					
Yes No	705 95	62 (8.79%) 14 (14.73%)	0.098	1.792	(0.960-3.346)
Drinking water system (n = 800)					
Yes No	344 456	13 (3.77%) 63 (13.81%)	0.000	4.08	(2.207-7.547)
Pets (n = 800)					
Yes No	80 720	11 (13.75%) 65 (9.03%)	0.222	1.60	(0.809-3.188)

## DISCUSSION

The present study determined the prevalence of *Giardia lamblia* infection among children in different districts of Punjab, Pakistan, and the potential risk factors associated with occurrences of giardiasis. This type of epidemiological study has routinely been conducted by using direct examination under a microscope or by using an immunochromatographic test (ICT), because the latter method is more cost-effective and less time-consuming. However, the low sensitivity of this test results in inaccurate data regarding the prevalence of this disease.

In the current study, direct examination under a microscope and the enzyme-linked immunosorbent assay (ELISA) were used in combination to detect the current prevalence of giardiasis. The overall prevalence of *Giardia lamblia* was found to be 9.5%. On the basis of area, the difference in the prevalence of *Giardia* was found to be non-significant, which is an indication that the disease is equally prevalent in geographically different districts. The results from our study greatly resemble the findings from other studies of 11.8% prevalence in Pakistan,^14^ 9% in Kabul^6^ and 6.8% in Portugal.^9^ Much higher levels of *Giardia* occurrence were observed among Afghan refugees (37.7%)^12^ and in Guatemala (43.8%).^15^ This variation in the prevalence of *Giardia lamblia* is probably due to differences in socioeconomic level between countries. Prevalences range from 2% to 7% in industrial countries and reach up to 40% in developing countries.^16^

The current study revealed that the prevalence of *Giardia* infection was 11.11% among children aged 0-5 years and 5.97% among those aged 6-10 years. These results were in accordance with previous findings from Julio et al.^9^ and Baido et al.^17^ A higher level of prevalence (31.9%) was reported in Russia among children aged 0-5 years.^18^ This may have been due to lack of acquired immunity among these children.^19^ The rate of positivity for *Giardia* in our study was almost equal between the sexes, and this was also seen in several previous studies.^17^^,^^20^^,^^21^ The presence of intestinal parasites in children results from some constant factors like food quality, water supply, personal and community hygiene, climate, sanitation conditions, proximity to domestic and wild animals and socioeconomic condition.^22^

The results from the present study revealed that the lower the mother’s educational level was, the higher the risk of *Giardia lamblia* infection. This finding is strengthened by the results from previous studies conducted in Pakistan,^14^ Malaysia,^23^ Tehran^24^ and Mexico.^25^ Similarly, it was also observed that the father’s educational level was inversely related to the risk of *Giardia lamblia* infection, and this is also supported by findings from previous research.^9^ This might be due to the fact that the father’s educational level is also reflected in socioeconomic status, such that lower status leads to poorer hygiene and sanitary conditions.

People living in rural areas showed higher levels of *Giardia intestinalis* infection than what was seen among people living in urban areas. This is also supported by data from previous studies, which showed that people living in rural areas had three times more chance of having giardiasis.^25^^,^^26^ Likewise, there was an inverse relationship between presence of a sewerage system and occurrence of *Giardia* infections. Similar results have also been recorded in many other studies.^9^^,^^26^ The reasons for this finding might be lack of sanitary and hygiene facilities in rural areas, as compared with urban areas, along with differences in awareness regarding the disease.

Drinking water is one of the major necessities of life and, if contaminated, it is a potential cause of many bacterial and parasitic diseases. In the present study, it was found that the prevalence of *Giardia lamblia* is greatly elevated among children who used untreated/unfiltered water, compared with the prevalence among those who used filtered or treated water for drinking purposes. The chances of infection if untreated water is used are four times greater.^9^^,^^26^ Attendance at day care centers among the children, unavailability of bathrooms and having pets at home increased the prevalence of *Giardia lamblia* infection among the children in our study, but these results were not statistically significant.

## CONCLUSION

The findings from this study showed that the prevalence of *Giardia lamblia* is still high and is an issue of public health concern. The factors strongly associated with occurrences of giardiasis were the parents’ educational level, lack of a bathroom, lack of sewerage facilities and unsafe drinking water. These findings indicate that improving these factors will have a positive impact on the wellbeing of Pakistani children. Further detailed studies at national level are needed regarding the epidemiology and burden of giardiasis, and the financial losses that it causes, in order to devise better control measures.
